# Non-linear Equation using Plasma Brain Natriuretic Peptide Levels to Predict Cardiovascular Outcomes in Patients with Heart Failure

**DOI:** 10.1038/srep37073

**Published:** 2016-11-15

**Authors:** Hiroki Fukuda, Hideaki Suwa, Atsushi Nakano, Mari Sakamoto, Miki Imazu, Takuya Hasegawa, Hiroyuki Takahama, Makoto Amaki, Hideaki Kanzaki, Toshihisa Anzai, Naoki Mochizuki, Akira Ishii, Hiroshi Asanuma, Masanori Asakura, Takashi Washio, Masafumi Kitakaze

**Affiliations:** 1 Department of Cardiovascular Medicine, National Cerebral and Cardiovascular Center, 5-7-1 Fujishirodai, Suita, Osaka, Japan; 2 Department of Cell Biology, National Cerebral and Cardiovascular Center, 5-7-1 Fujishirodai, Suita, Osaka, Japan; 3 Department of Clinical Research and Development, National Cerebral and Cardiovascular Center, 5-7-1 Fujishirodai, Suita, Osaka, Japan; 4 The institute of Scientific and Industrial Research, Osaka University, 1-1 Yamadaoka, Suita, Osaka, Japan; 5 Department of Cardiovascular Medicine, Kyoto Prefectural University of Medicine, Kyoto, Japan

## Abstract

Brain natriuretic peptide (BNP) is the most effective predictor of outcomes in chronic heart failure (CHF). This study sought to determine the qualitative relationship between the BNP levels at discharge and on the day of cardiovascular events in CHF patients. We devised a mathematical probabilistic model between the BNP levels at discharge (y) and on the day (t) of cardiovascular events after discharge for 113 CHF patients (Protocol I). We then prospectively evaluated this model on another set of 60 CHF patients who were readmitted (Protocol II). P(t|y) was the probability of cardiovascular events occurring after >t, the probability on t was given as p(t|y) = −dP(t|y)/dt, and p(t|y) = pP(t|y) = αy^β^P(t|y), along with p = αy^β^ (α and β were constant); the solution was p(t|y) = αy^β^ exp(−αy^β^t). We fitted this equation to the data set of Protocol I using the maximum likelihood principle, and we obtained the model p(t|y) = 0.000485y^0.24788^ exp(−0.000485y^0.24788^t). The cardiovascular event-free rate was computed as P(t) = 1/60Σ_i=1,…,60_ exp(−0.000485y_i_^0.24788^t), based on this model and the BNP levels y_i_ in a data set of Protocol II. We confirmed no difference between this model-based result and the actual event-free rate. In conclusion, the BNP levels showed a non-linear relationship with the day of occurrence of cardiovascular events in CHF patients.

Because chronic heart failure (CHF) is one of the most common fatal diseases and has an increasing prevalence worldwide^1^, its severity must be correctly diagnosed for the appropriate treatment to be implemented. Because it has been shown that brain natriuretic peptide (BNP) is linked to the pathophysiology of HF^2^,^3^ and that higher plasma BNP levels qualitatively predict more frequent clinical events, such as hospitalization or cardiovascular death, clinicians refer to the plasma BNP levels when diagnosing or treating CHF patients^4^,^5^. Plasma BNP levels have been recognized to correspond to the severity of CHF because of the peptide secreted from cardiac cells in response to wall stress of the heart^6^,^7^,^8^. However, BNP is known to be cardioprotective^9^,^10^. Therefore, BNP functions to improve cardiovascular function and reflect cardiovascular dysfunction, which suggests that it is intricately and complexly linked to the pathophysiology of CHF. However, there has not been a clear consensus on whether the plasma BNP levels quantitatively predict the time of cardiovascular events in CHF patients. Furthermore, it is unclear whether the extent of BNP-level changes quantitatively predicts the interval to the occurrence of cardiovascular events. For subjects with stable CHF, increased risk is evident in patients with a BNP level of at least 100 pg/mL; every increase of 100 pg/mL is associated with a 35% increase in risk^11^,^12^. However, the probability that BNP levels of 100–200 pg/mL have a comparable tendency to cause cardiovascular events as BNP levels of 900–1,000 pg/mL is questionable. Based on this hypothesis, we aimed to construct an exquisite model to establish the link between the plasma BNP levels and the day of re-hospitalization because of the worsening of HF or cardiovascular death in patients with HF.

Using our database of patients who were admitted because of acute decompensated heart failure (ADHF) and were discharged as stable CHF after sufficient treatment, we performed this study with the following objectives: (1) to theoretically derive a probability formula that correlates the plasma BNP levels at discharge and the day of hospitalization or cardiac death in patients with stable CHF; (2) to obtain an actual probability model by fitting the theoretical probability formula to the clinical data of the retrospectively enrolled patients; and (3) to prospectively test whether the cardiovascular event-free rate using this model in CHF patients was consistent with the actual cardiovascular event-free rate in the patients.

## Methods

### Study protocols

In Protocol I, we retrospectively enrolled CHF patients to constitute an actual probability model that would provide the day of cardiovascular events based on the plasma BNP levels alone. In Protocol II, we prospectively tested whether the expected date, as determined by the probability model, was consistent with the actual day of cardiovascular events.

### Protocol I

From our database of CHF patients, 113 ADHF patients who were admitted because of worsening CHF and were discharged after improvement of HF to New York Health Association (NYHA) I or II between January 2007 and December 2008 were retrospectively enrolled. The follow-up period was until December 2014. For patients who were admitted to the hospital several times during the follow-up period, the earliest date of hospitalization was recorded. CHF was diagnosed by an expert team of cardiologists using the Framingham criteria^13^. The time of the discharge was determined by the expert team of cardiologists in charge of the HF department. Discharge was recommended when the patients presented no signs of decompensation, such as NYHA functional class <3, rales, and gallop rhythm; when their blood pressure was stable; and when there was an improvement in renal function after optimal treatment following international guidelines^14^. In each patient, the plasma BNP level at discharge was recorded. Similarly, the first day of occurrence of cardiovascular events was recorded. A cardiovascular event was defined as re-hospitalization because of worsening HF or cardiovascular death. An independent group of physicians in the emergency room at our institute made the decision on re-hospitalization when the CHF of the patients progressed and worsened; they also analysed the primary cause of re-hospitalization or death. We fitted our theoretical probability model, which is described below, to the data on the BNP levels at discharge and the day of the cardiovascular events to approximate the probability of cardiovascular events based on these BNP levels.

### Protocol II

We prospectively enrolled 60 ADHF patients who were admitted because of worsening HF and were discharged after improvement of HF to NYHA I or II between May 2013 and December 2015. We followed-up these patients until the end of 2015. The primary endpoint was the day of the first re-hospitalization because of cardiovascular causes or cardiovascular death. An independent group of physicians in the emergency room of our institute made a decision on re-hospitalization when the CHF of the patients progressed and worsened; they also analysed the primary cause of re-hospitalization or death. We prospectively tested whether the predictive curves of the cardiovascular event-free rate produced by the mathematical model were significantly close to the Kaplan-Meier curves for the actual cardiovascular event-free rate during the follow-up period.

### Constitution of a mathematical model to link plasma BNP levels and the day of cardiovascular events for Protocols I and II

We assumed that the CHF severity in most patients did not significantly change after discharge and proportionally reflected the daily probability of cardiovascular events (p). We chose the plasma BNP levels as the most valuable index for assessing CHF severity because BNP is produced according to the progression of CHF and because BNP clearance from the blood is proportional to the extent of cardioprotection. On the basis of these assumptions, we estimated that the daily production and consumption of plasma BNP were proportional to the power of p (p^γ^) and the plasma BNP level at discharge from the hospital (y), respectively. Because daily production (∝p^γ^) and consumption (∝y) were considered to be almost equal in a given patient, p^γ^ = θy holds. This equation was rewritten as:


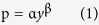


where α = θ^1/γ^ and β = 1/γ were constant.

Second, P(t|y) was designated as the probability that cardiovascular events did not occur before an interval (t) having elapsed from discharge under a given y. Then, p(t|y) was designated as the probability of cardiovascular events on day t and was equal to the daily reduction of P(t|y) (i.e., −dP(t|y)/dt). At the same time, p(t|y) was equal to the probability of the first occurrence of a cardiovascular event on day t, denoted as pP(t|y), which is the probability that the event did not occur before day t but occurred on day t. Accordingly, we obtained the following differential equation:


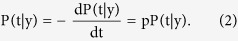


Because all patients were alive at discharge under a given y, P(0|y) = 1 holds. Then, we derived the following solution of this differential equation with Equation (1):


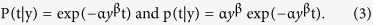


Given a data set of Protocol I, DI = {(y*i*,t*i*)|i = 1,…,N}, the log-likelihood under this solution was represented as follows:


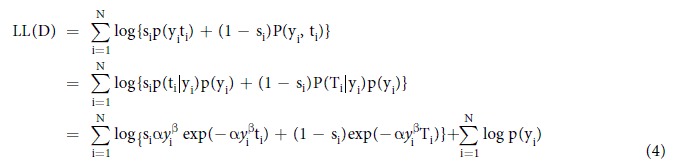


where s_i_ was equal to 1 if the clinical event of patient i occurred within the time span of observation in DI; otherwise, s_i_ was equal 0. T_i_ was the elapsed day at the close of the observation for patients with s_i_ = 0. p(y, t) is the probability of observing a patient with a BNP level of y at discharge and a cardiovascular event at day t in the data set. Similarly, P(y, T) is the probability of observing a patient with a BNP level of y at discharge and a cardiovascular event after day T in the data set. p(y) is the distribution of y in the data set. Because the second term on the last line did not depend on parameters α and β, we searched for the combination of these parameters that maximized the first term on the data set DI by a standard non-linear optimization algorithm named the Nelder–Mead Simplex Method^15^.

Under these optimum parameters obtained by DI of Protocol I, the model-based event-free rate (P(t)) at t for a BNP data set of Protocol II, DII_y_ = {y_i_|i = 1, …, M}, was computed using an approximation of p(y) by its data distribution in DII_y_: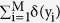, where δ(•) is Dirac’s delta function. Because P(t) was equivalent to the probability that a cardiovascular event occurred after day t, it was computed as follows:


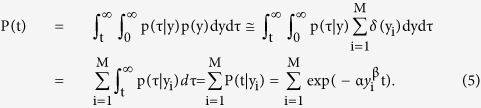


Moreover, the model obtained by DI enabled us to compute the event-free rate curve P(t) for a group of patients with BNP levels in a specific interval (y_l_, y_u_) at discharge in DII_y_. This was performed by limiting the distribution p(y) into (y_l_, y_u_), *i.e.*, limiting the patients to those having BNP levels in (y_l_, y_u_) in DII_y_ as 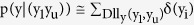 where the summation was taken over DII_y_(y_l_, y_u_) = {y_i_|y_l_ < y_i_ < y_u_ in DII_y_}. Thus, we showed an event-free rate curve P(t|(y_l_, y_u_)) for the patient group as:


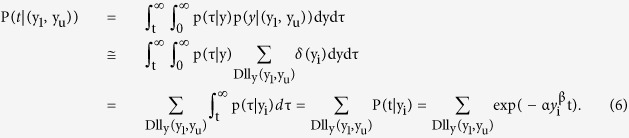


### Ethics Statement

Both Protocols I and II were approved by the National Cerebral and Cardiovascular Centre Research Ethics Committee. For Protocol I, the committee decided that the acquisition of informed consent from the 113 subjects was not required, according to the Japanese Clinical Research Guidelines and because this was a retrospective observational study. Instead, we made a public announcement on both the Internet homepage of our institution and the bulletin boards of our outpatient and inpatient clinics, in accordance with the request of the ethics committee and the guidelines. In Protocol II, written informed consent was obtained from all 60 subjects (UMIN000018691). This was a clinical study that enrolled patients with CHF, and all methods were performed in accordance with the relevant guidelines and regulations.

### Statistical Analysis

Normally distributed data are expressed as the mean ± standard deviation; other values were reported as median and interquartile range (IQR). We performed the goodness-of-fit test and used the coefficient of determination as a measure to assess the significant relationships between the predictive curves and actual Kaplan-Meier curves of the cardiovascular event-free rate. The differences in the predictive curves among the different BNP levels were tested using the Wilcoxon signed-rank test. We estimated the error bounds of the parameters α and β by applying the standard bootstrap sampling^16^ to the data set DI of Protocol I, and we added the upper and lower 2σ bound lines of the model based Kaplan-Meier curves obtained in Protocol II. All tests were two-tailed, and P < 0.05 was considered significant. All analyses were performed using JMP software for Windows (version 8.0.2, SAS Inc., Cary, NC, USA).

## Results

**Figure 1:**
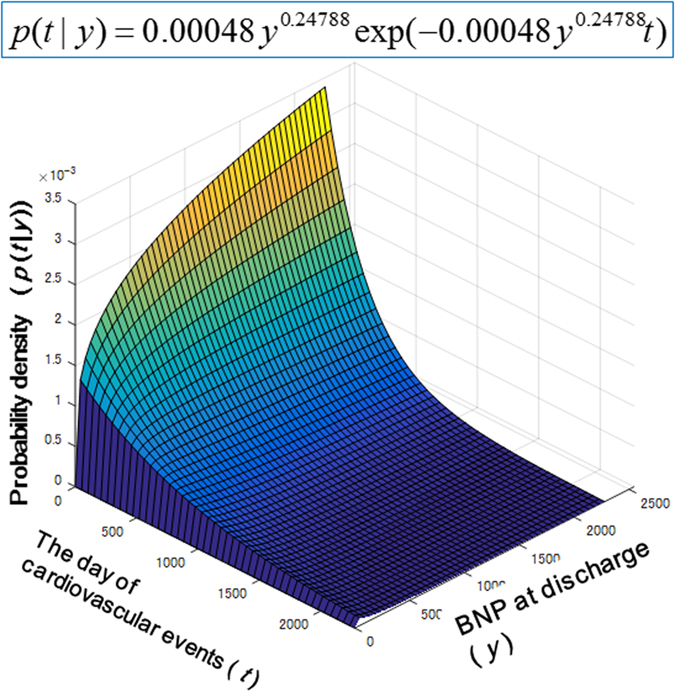
Fitting of the actual data on the plasma BNP levels and the day of cardiovascular events to the theoretical equation model for patients with ADHF. This shows the conditional probability of the elapsed date at a clinical event after discharge (t) under a given BNP level at discharge (y).

In Protocol I, a total of 113 HF patients (71 men and 42 women, median age of 67 years) were retrospectively enrolled in this study (Table 1). All patients were in NYHA functional class I or II upon discharge. The data on the plasma BNP levels at discharge were used to obtain a probability model for the day of cardiovascular events because it approximated the function of plasma BNP. We fitted the model to the data on the plasma BNP levels at discharge and the day of cardiovascular events by using the maximization of the aforementioned logarithmic likelihood LL(D). We obtained the parameters of α = 0.000485 and β = 0.24788 to characterize the daily probability of the cardiovascular event as equation (1) at the optimum. Accordingly, we obtained the probability model p(t|y) = 0.000485y^0.24788^ exp(−0.000485y^0.24788^t) (Fig. 1).

**Figure 2:**
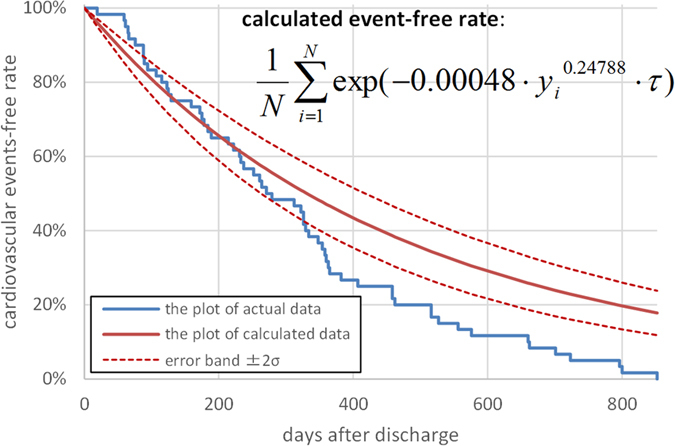
The Kaplan-Meier plots of calculated and actual cardiovascular event-free rate in Protocol II. The actual cardiovascular events started slightly later than the calculated events and ended earlier than the calculated events; however, the goodness-of-fit model found that KM and predictive curves were significantly close, and the coefficient of determination was 0.92.

**Figure 3:**
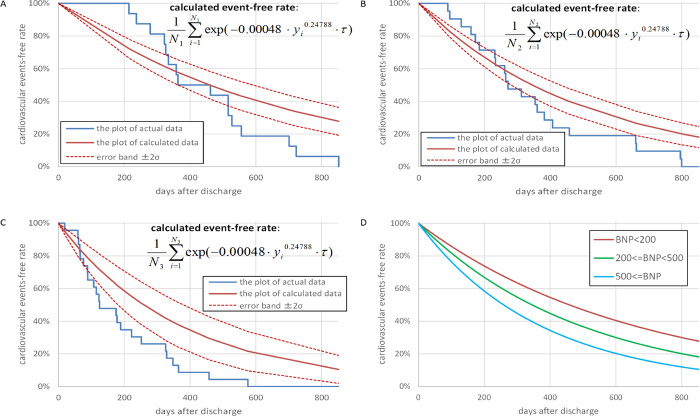
The Kaplan-Meier plots of calculated and actual cardiovascular event-free rate in patients with plasma BNP levels ≤ 200 pg/mL (**A**), 200 pg/mL <BNP ≤ 500 pg/mL (**B**) and BNP levels>500 pg/mL (**C**). These two lines in each group are significantly close, and the coefficients of determination in **A**–**C** are 0.94, 0.90 and 0.96, respectively. Figure (**D**) shows Kaplan-Meier plots of three calculated cardiovascular events-free rates in the sets of the patients with the plasma BNP levels ≤ 200 pg/mL, 200 pg/mL <BNP ≤ 500 pg/mL and BNP levels>500 pg/mL.

For Protocol II, the patients’ characteristics are shown in Table 2. Figure 2 shows the Kaplan-Meier plots calculated by using the actual data and the model for cardiovascular event-free periods. Both lines are significantly close to each other (R^2^ = 0.92). The upper and lower 2σ bound lines of the model plot did not completely overlap with the actual Kaplan-Meier curve because the actual Kaplan-Meier curves also fluctuated due to their statistical uncertainty. Figure 3A–C show the Kaplan-Meier plots of the actual data and the model for the cardiovascular event-free period in each group, with plasma BNP levels ≤ 200 pg/mL (n = 16), 200 pg/mL <BNP ≤ 500 pg/mL (n = 21) and BNP levels > 500 pg/mL (n = 23). The 2σ bounds of the model-based curves are also provided. In each group, both lines are significantly close to each other (R^2^ = 0.94, 0.90 and 0.95 for Fig. 3A–C). Figure 3D depicts the Kaplan-Meier plots of the model-based event-free rates and shows that the plots become steeper when the BNP levels increase.

## Discussion

Plasma BNP levels are known to be related to the probability of cardiovascular events, such as hospitalization or cardiovascular death. However, there is no clear consensus on whether the plasma BNP levels quantitatively predict the time of occurrence of cardiovascular events. In the present study, we constituted a theoretical probability model to estimate the time of cardiovascular event occurrence based on the plasma BNP levels at discharge in CHF patients who were admitted to our hospital because of ADHF. We provided a non-linear probability model, p(t|y) = 0.000485y^0.24788^ exp(−0.000485y^0.24788^t), and a model-based formula of the event-free rate, 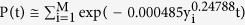, by fitting the actual data on the BNP levels and the day of cardiovascular events. We further validated the accuracy and reproducibility of this model in a prospective observational study and found that the model provided a plausible probability of a given BNP level to assess the time of occurrence of cardiovascular events. As shown in Fig. 3, the calculated and actual cardiovascular event-free rates are well correlated with each other, although the times of the beginning and end of cardiovascular events were slightly separate. This figure is comparable to the data from the Seattle Heart Failure Model^17^; however, our model is derived from only the BNP levels at discharge based on the pathophysiological assumption of BNP metabolism and by fitting the obtained equation to the actual data and validating the obtained equation using CV and another ADHF cohort, whereas Levy *et al.* obtained the predicted equation by fitting the survival to many factors using multivariate analysis and validating it with another cohort of CHF^17^.

BNP is produced in cardiomyocytes by both ventricular stress and excess neurohumoral factors^6^,^7^,^8^. Because the LV mass is larger than the RV mass, the plasma BNP levels primarily reflect LV dysfunction. Plasma BNP appears to be cardioprotective by increasing cyclic GMP levels and activating G kinase^10^, but it also increases with worsening HF^18^; these facts provide evidence that increases in the BNP levels may precisely predict the occurrence of cardiovascular events in patients with HF. Indeed, the plasma BNP levels were shown to decrease after treatment modalities that are known to improve the clinical outcomes of patients with CHF^19^. This suggests that BNP not only is a biomarker of the severity of cardiac dysfunction but also is linked to cardioprotection. Therefore, the BNP levels may precisely predict the clinical outcomes in CHF patients.

These characteristics of BNP are different from those of other simple biological or physiologic markers of HF. For example, LV ejection fraction (LVEF) is one of the physiologic markers of HF, but treatment modalities, such as inotropic agents, that increase LVEF are not necessarily beneficial for HF. However, BNP is important for a biomarker-guided study^19^, which suggests that it is a more important biomarker for the prediction of cardiovascular events.

Why can the plasma BNP levels at discharge predict the probability of cardiovascular events? It does not seem to be convincing that a one-time measurement of the plasma BNP level at discharge in each HF patient can predict the day of occurrence of cardiovascular events because the BNP levels may fluctuate weekly or monthly during outpatient follow-up, despite the constant intake of medications. These changes in the BNP levels may be attributed to daily changes in hemodynamic parameters, such as heart rate or blood pressure; excessive intake of water or salt; or temporal sympathetic activation in a patient^20^. These facts suggest that the BNP levels at discharge cannot determine the fate of the CHF patients. However, CHF patients who are discharged from the hospital receive the ideal medications, medical apparatus, water/salt balance, and haemodynamics; this implies that patients are in the best condition at discharge, thus providing the lowest value of BNP levels for that patient. Indeed, in this study, the plasma BNP level at admission decreased from 917 to 351 pg/mL after comprehensive treatment for ADHF (Table 1). Such BNP levels may be considered to represent the best clinical condition of the patient and may reflect the true severity of CHF and the probability of cardiovascular events.

Indeed, reports have shown that the pre-discharge BNP or NT-pro BNP levels that were measured only during hospitalization predicted clinical outcomes^21^,^22^. This BNP value at the best clinical condition may affect the probability of the clinical outcomes. Of course, the actual and baseline cardiac function may worsen during follow-up; we cannot deny this possibility, and this deviation may cause the actual day of occurrence of cardiovascular events to deviate from the estimated days.

BNP in plasma has been used as a sole biomarker for CHF and is recognized to precisely predict cardiovascular events. However, specialized medical care has been focused because many clinical, social, and genomic factors are involved in patients with CHF^23^. However, at the same time, we should seek the most important factor to ascertain the disease and investigate the precise relationship between that factor and the clinical outcomes. This method of investigation that uses BNP as a biomarker for CHF may also contribute to the choice of an appropriate medicine.

**Figure 4:**
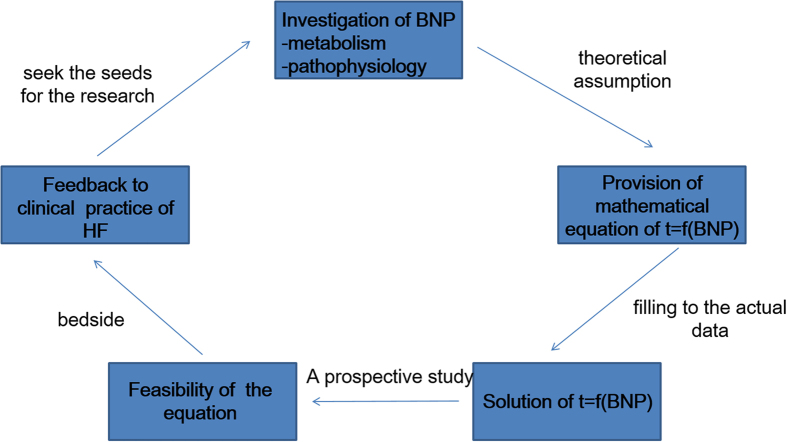
The schema and procedure to provide the mathematical probabilistic model between plasma BNP levels and the day of cardiovascular events. BNP, brain natriuretic peptide; HF, heart failure.

The most important information of the present study was that the increase in the risk for re-hospitalization was not proportional or linear to the increase in BNP levels for subjects with stable CHF following ADHF, even if the subsequent BNP levels were more than 100 pg/mL, although every 100 pg/mL rise was associated with a 35% increase in risk for subjects with stable CHF. The probability of occurrence of clinical outcomes in patients with CHF was related to the BNP levels at a power of approximately 1/4. We do not know how the changes in the BNP levels correspond to the quantitative improvement or worsening of CHF, and we believe that one of the advantages of the present study is that we can propose such an equation to yield quantitative meanings for the BNP values. It would be better to transform the BNP levels to 1/BNP^0.25^ to obtain an index of CHF severity from the viewpoint of clinical events. The other impact of the present study is how to employ mathematical science in clinical practice. Figure 4 shows the scenario of the present study, which aimed to both provide a theoretical equation that can be fitted to actual retrospective clinical data and evaluate the equation in a prospective study.

### Limitations

The present study had several limitations. First, because this was a single-centre study, the number of patients was limited, particularly that of subjects with ADHF. Second, the quantity of data was somewhat small. However, we obtained the probability equation for 113 patients in a retrospective study and provided evidence for the plausibility of this equation in 60 patients in a prospective study. In this sense, we suggest testing the present hypothesis using multicentre trials.

## Conclusions.

Our study showed that the plasma BNP levels non-linearly predicted the day of occurrence of clinical events in patients with CHF. By using our equation, the impact of BNP levels on cardiovascular outcomes may be quantitatively evaluated.

## Additional Information

**How to cite this article**: Fukuda, H. *et al.* Non-linear Equation using Plasma Brain Natriuretic Peptide Levels to Predict Cardiovascular Outcomes in Patients with Heart Failure. *Sci. Rep.* **6**, 37073; doi: 10.1038/srep37073 (2016).

**Publisher’s note:** Springer Nature remains neutral with regard to jurisdictional claims in published maps and institutional affiliations.

## Figures and Tables

**Table 1:** Patient characteristics of the Protocol I.

	Population (n = 113)
Age, yrs	63.6 ± 16.2
Male	70 (62)
Cause of ADHF	
Cardiomyopathy	45 (40)
Hypertensive heart disease	13 (12)
Ischemic heart disease	12 (11)
Valvular heart disease	26 (23)
Cardiac sarcoidosis	4 (3)
Myocarditis	1 (1)
Others	12 (11)
Comorbidity	
Hypertension	45 (40)
Diabetes mellitus	32 (28)
Hyperlipidemia	25 (22)
Chronic AF	25 (22)
Signs at admission	
Elevated jugular venous pressure	55 (49)
S_3_ gallop	71 (63)
Lower extremity edema	63 (56)
NYHA functional class: III/IV	75 (66)/38 (34)
Baseline characteristics at admission/at discharge	
Heart rate, beats/min	83 ± 26/66 ± 10
Systolic BP, mmHg	120 ± 31/105 ± 15
Diastolic BP, mmHg	69 ± 17/59 ± 7
Body weight, kg	58.8 ± 13.4/53.3 ± 12.1
Laboratory factors at discharge	
Hemoglobin, g/dl	12.2 ± 2.1
Blood urea nitrogen, mg/dl	26.0 ± 11.9
Creatinine, mg/dl	1.13 ± 0.56
Sodium, mEq/L	136.8 ± 4.0
Uric acid, mg/dl	7.2 ± 2.2
C-reactive protein, mg/dl	0.5 ± 1.3
BNP at admission/at discharge, median (IQR), pg/ml	626 (276–1123)/244 (125–397)
Echocardiographic factors at admission/at discharge	
Left ventricular end-diastolic dimension, mm	58.8 ± 13.2/58.9 ± 12.5
Left ventricular end-systolic dimension, mm	46.5 ± 15.4/46.3 ± 15.7
Fractional shortening, %	22.4 ± 12.2/23.2 ± 12.6
Left atrial diastolic dimension, mm	51.0 ± 9.2/48.4 ± 8.6
Pressure across tricuspid valve, mmHg	38.5 ± 15.0/28.2 ± 12.3
Medication at admission	
Use of dopamine	6 (4)
Use of dobutamine	28 (25)
Use of phosphodiesterase inhibitor	16 (14)
Use of hANP	23 (20)
Use of nitroglycerin	14 (12)
Use of intravenous diuretics	37 (33)
Medication at discharge	
ACEi	46 (41)
ARB	33 (29)
Beta-blockers	80 (71)
Digitalis	38 (34)
Diuretics	99 (88)

Values are mean ± SD or n (%) unless otherwise indicated.

IQR, interquartile range; ADHF, acute decompensated heart failure; AF, atrial fibrillation; NYHA, New York Heart Association; BNP, brain natriuretic peptide; BP, blood pressure; hANP, human atrial natriuretic peptide; ACEi, angiotensin converting enzyme inhibitor; ARB, angiotensin II receptor blocker.

**Table 2:** Patient characteristics of the Protocol II.

	Population (n = 60)
Age, yrs	65.9 ± 17.4
Male	38 (63)
Cause of ADHF	
Cardiomyopathy	26 (43)
Hypertensive heart disease	5 (8)
Ischemic heart disease	10 (17)
Valvular heart disease	10 (17)
Cardiac sarcoidosis	1 (2)
Myocarditis	1 (2)
Others	7 (12)
Comorbidity	
Hypertension	29 (48)
Diabetes mellitus	21 (35)
Hyperlipidemia	20 (33)
Chronic AF	26 (43)
Signs at admission	
Elevated jugular venous pressure	28 (47)
S_3_ gallop	34 (57)
Lower extremity edema	41 (68)
NYHA functional class: III/IV	35 (58)/25 (42)
Baseline characteristics at admission/at discharge	
Heart rate, beats/min	82 ± 19/66 ± 9
Systolic BP, mmHg	123 ± 23/107 ± 20
Diastolic BP, mmHg	70 ± 16/61 ± 17
Body weight, kg	60.8 ± 15.2/56.2 ± 14.5
Laboratory factors at discharge	
Hemoglobin, g/dl	12.1 ± 2.4
Blood urea nitrogen, mg/dl	39.0 ± 27.4
Creatinine, mg/dl	1.09 ± 0.40
Sodium, mEq/L	138.6 ± 5.3
Uric acid, mg/dl	6.7 ± 2.4
C-reactive protein, mg/dl	1.1 ± 2.0
BNP at admission/at discharge, median (IQR), pg/ml	963 (754–1366)/377 (198–816)
Echocardiographic factors at admission/at discharge	
Left ventricular end-diastolic dimension, mm	61.0 ± 13.5/62.0 ± 14.3
Left ventricular end-systolic dimension, mm	51.2 ± 16.6/51.7 ± 17.3
Fractional shortening, %	17.8 ± 12.2/18.3 ± 12.2
Left atrial diastolic dimension, mm	51.7 ± 7.8/47.8 ± 7.6
Pressure across tricuspid valve, mmHg	40.1 ± 8.5/30.3 ± 6.8
Medication at admission	
Use of dopamine	3 (5)
Use of dobutamine	12 (20)
Use of phosphodiesterase inhibitor	10 (17)
Use of hANP	21 (35)
Use of nitroglycerin	15 (25)
Use of intravenous diuretics	23 (38)
Medication at discharge	
ACEi	28 (47)
ARB	9 (15)
Beta-blockers	48 (80)
Digitalis	18 (30)
Diuretics	55 (92)

Values are mean ± SD or n (%) unless otherwise indicated.

IQR, interquartile range; ADHF, acute decompensated heart failure; AF, atrial fibrillation; NYHA, New York Heart Association; BNP, brain natriuretic peptide; BP, blood pressure; hANP, human atrial natriuretic peptide; ACEi, angiotensin converting enzyme inhibitor; ARB, angiotensinII receptor blocker.
